# Magneto-Dielectric Behaviour of M-Type Hexaferrite/Polymer Nanocomposites

**DOI:** 10.3390/ma11122551

**Published:** 2018-12-14

**Authors:** Aikaterini Sanida, Sotirios Stavropoulos, Thanassis Speliotis, Georgios C. Psarras

**Affiliations:** 1 Smart Materials & Nanodielectrics Laboratory, Department of Materials Science, School of Natural Sciences, University of Patras, 26504 Patras, Greece; ksanida@upatras.gr (A.S.); stavropoulos@upatras.gr (S.S.); 2 Institute of Nanoscience and Nanotechnology, NCSR “Demokritos”, Aghia Paraskevi, 15310 Athens, Greece; t.speliotis@inn.demokritos.gr

**Keywords:** barium ferrite, strontium ferrite, ferromagnetic nanocomposites, dielectric relaxations, magnetization, zero field cooled, field cooled, magnetic transition

## Abstract

In the present study two sets of nanocomposites consisting of an epoxy resin and BaFe_12_O_19_ or SrFe_12_O_19_ nanoparticles were successfully developed and characterized morphologically and structurally via scanning electron microscopy and X-ray diffraction spectra. The dielectric response of the nanocomposites was investigated by means of broadband dielectric spectroscopy and their magnetic properties were derived from magnetization tests. Experimental data imply that the incorporation of the ceramic nanoparticles enhances significantly the dielectric properties of the examined systems and their ability to store electrical energy. Dielectric spectra of all systems revealed the presence of three distinct relaxation mechanisms, which are attributed both to the polymer matrix and the nanoinclusions: Interfacial polarization, glass to rubber transition of the polymer matrix and the re-orientation of small polar side groups of the polymer chain. The magnetic measurements confirmed the ferromagnetic nature of the nanocomposites. The induced magnetic properties increase with the inclusion of hexaferrite nanoparticles. The nanocomposites with SrFe_12_O_19_ nanoparticles exhibit higher values of coercive field, magnetization, magnetic saturation and remanence magnetization. A magnetic transition was detected in the ZFC/FC curves in the case of the BaFe_12_O_19_/epoxy nanocomposites.

## 1. Introduction

Ferrites belong in a large and well-established class of ceramic magnetic materials with a wide range of technological applications [1]. Ferrites are classified into two major types, the soft magnets characterized by domain walls which easily can be moved by an externally applied magnetic field and the hard magnets that are chemical compounds of metal oxides with strong magnetic properties, which are ideal for permanent magnets [2]. The M-type hexaferrites MFe_12_O_19_ (M = Ba, Sr or Pb) are important ferromagnetic oxides and have traditionally be used as permanent magnets in applications for dielectric media because of their high values of magneto-crystalline anisotropy and saturation magnetization. Because of their chemical stability, high intrinsic coercivity, large crystal anisotropy, high electrical resistivity, low cost, enhance resistance to the heat and high corrosion resistance, ferrites can be employed as components in a variety of microwave and high frequency electronic devices, used in magnetic recording media, communication, generation and distribution of electric energy, automotive and medical equipment [1,2,3,4,5,6,7]. Even though hexaferrites, have long been studied and become established materials, there are still several areas that are not completely defined and understood and new exciting properties are being discovered and opportunities are developed [6,8,9,10].

Polymers exhibit several advantages including easy processing and forming, thermomechanical stability, high dielectric breakdown strength and low cost. This is the main reason why many research efforts have focused on ceramic/polymer nanocomposites. Such composite materials can be moulded into complex shapes and they are suitable for adaptive devices where flexibility and elasticity are an additional and important parameter. Most studies to date have shown that polymer properties (such as mechanical, electrical, thermal, optical etc.) can be controlled by embedding suitable reinforcing nanoparticles [11,12]. Among others, ferrite nanoinclusions have been embedded in polymer matrix. Although the available studies are scarce and mostly concern physical and mechanical properties [13,14], it has been shown that a fine distribution of the magnetic filler in a polymer matrix influences significantly the conductivity, dielectric [13,15,16,17,18] and magnetic properties [19,20,21] of the systems, via the effective volume fraction of the nanoparticles. In the electronics industry, epoxy resins being excellent electrical insulators are the primary resin used in integrated circuits, transistors, hybrid circuits and printed circuit boards protecting electrical components from short circuiting, dust and moisture [22,23]. Additionally, flexible epoxy resins are used for potting transformers and inductors. 

In this study, series of nanocomposites constituted of an epoxy resin acting as the matrix and two different hexaferrite nanoparticles (BaFe_12_O_19_ and SrFe_12_O_19_) as the reinforcing phase, have been prepared and studied varying the filler content. Ferrites in their single crystal or ceramic form have been studied as monolithic materials [9,24,25]. In contrast, scarce attention has been given to ferrite/polymer nanocomposites [14,26] although ferrite nanoparticles could be beneficial to a bunch of composite properties including mechanical, electrical and magnetic performance. The quality of the developed systems was investigated via scanning electron microscopy (SEM) and X-ray diffraction (XRD) spectra, while dielectric and magnetic response were studied by means of broadband dielectric spectroscopy (BDS) and magnetization measurements employing a SQUID magnetometer, respectively.

## 2. Materials and Methods

Two different series of ferromagnetic nanocomposites were prepared using commercially available materials. In particular, a low viscosity epoxy resin (Epoxol 2004 A) along with its curing agent (Epoxol 2004 B), provided by Neotex S.A., Athens, Greece and BaFe_12_O_19_, SrFe_12_O_19_ nanoparticles, supplied by Sigma Aldrich (Saint Louis, MO, USA), were employed. The size of nanoparticles, according to the supplier’s data sheet, is less than 100 nm.

The specimens manufacturing procedure included mixing of the epoxy prepolymer and curing agent in a 2:1 (*w*/*w*) ratio and the subsequent addition of various amounts of the ceramics nanopowder at room temperature, while stirring under ultrasonication for 10 min, in order to achieve homogeneous filler dispersion and to avoid agglomeration. Afterwards mixtures were poured into silicon moulds for curing. Initial curing took place at ambient for seven days, followed by post-curing for four hours at 100 °C. Manufactured specimens, were in the form of circular disk with diameter 36 mm and width 2 mm for dielectric measurements and in the form of cylinders with 6 mm and 5 mm diameter and height respectively for magnetic measurements. The filler concentration in the nanocomposites is expressed in parts per hundred resin per weight (phr).

The morphological characterization of the prepared samples was conducted by scanning electron microscopy (SEM) using an EVO MA-10 apparatus supplied by Carl Zeiss. For the structural investigation, X-ray diffraction (XRD) spectra were obtained via a Siemens diffractometer model Z500, by using Cu-Ka (λ = 1.54056 Å, 40 kV, 30 mA) in a wide range of Bragg angles 20–90°, for both the BaFe_12_O_19_ and SrFe_12_O_19_ nanopowders and the nanocomposites.

Dielectric characterization was carried out by means of broadband dielectric spectroscopy (BDS) in the frequency range of 10^−1^–10^7^ Hz using an Alpha-N Frequency Response Analyzer and temperature interval from 30 to 160 °C, with a temperature step of 10 °C, controlled by Novotherm System. The specimens were placed inside the dielectric cell BDS 1200 which is a parallel plate capacitor with two gold-plated electrodes and the experimental data were obtained automatically via suitable software (Windeta), by performing isothermal frequency scans with the amplitude of the time varying voltage being equal to 1 V in all cases. The experimental equipment and the software were supplied by Novocontrol Technologies.

The magnetic characterization was conducted by employing a superconducting quantum interference device (SQUID) magnetometer, supplied by Quantum Design. The maximum applied magnetic field was 50 kOe. The zero-field-cooled (ZFC) and field cooled (FC) processes were recorded at low magnetic fields in temperature range from 5 to 300 K. Prior of each run, specimens were demagnetized at 300 K by applying an oscillatory magnetic field and then cooled down in zero field to 5 K. At 5 K, a small magnetic field of the order of 100 or 1000 Oe was applied in the powder and the nanocomposites and the magnetization was measured as the sample was heated to 300 K. This procedure was denoted as the ZFC measurement. At 300 K, the small-applied magnetic field was kept as it is and then samples were cooled again to 2 K, with a subsequent recording of the magnetization. Such measurement is denoted as FC measurement.

## 3. Results

### 3.1. Morphological and Structural Characterization

**Figure 1:**
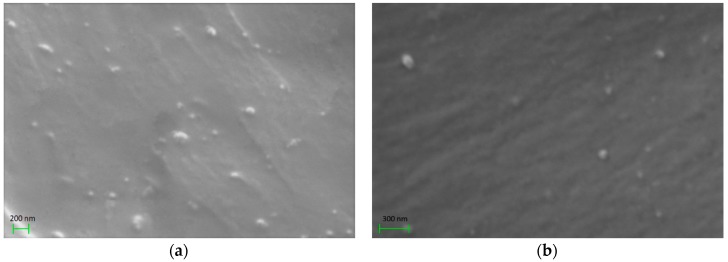
Scanning electron microscopy (SEM) images for the nanocomposites with 5 phr: (**a**) BaFe_12_O_19_; (**b**) SrFe_12_O_19_ content.

Figure 1 shows representative SEM images of the nanocomposites with 5 phr BaFe_12_O_19_ and SrFe_12_O_19_. The obtained SEM images revealed nanodispersions of the ceramic particles in the polymer matrix, while the formation of large aggregates or agglomerations has been avoided in both systems.

**Figure 2:**
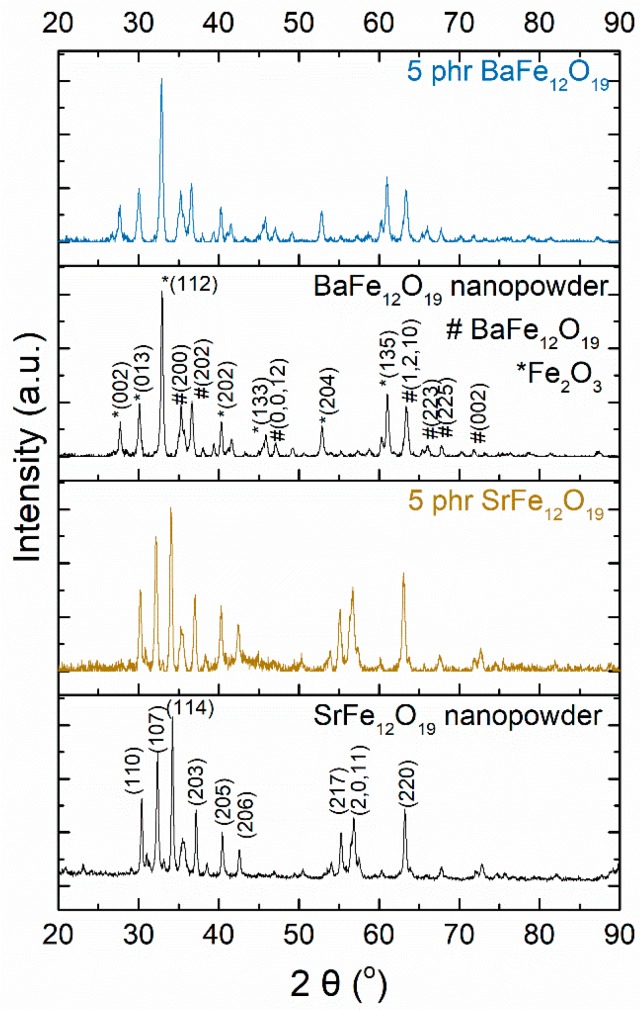
X-ray diffraction (XRD) spectra of the BaFe_12_O_19_ and SrFe_12_O_19_ nanopowders and their respective nanocomposites with 5 phr filler content.

XRD patterns of the BaFe_12_O_19_ and SrFe_12_O_19_ nanopowders and their respective nanocomposites with 5 phr filler content are illustrated in Figure 2. The diffraction peaks can be indexed to the typical magnetoplumbite-type (M-type) hexagonal structures of BaFe_12_O_19_ and SrFe_12_O_19_. XRD data revealed the coexistence of Fe_2_O_3_ [27] within both the BaFe_12_O_19_ nanoparticles and its respective nanocomposites, probably as a residue of the fabrication method of the ceramic nanopowder. In the SrFe_12_O_19_ nanopowder and the corresponding nanocomposites no characteristic peaks for other impurities have been observed.

### 3.2. Dielectric Characterization

**Figure 3:**
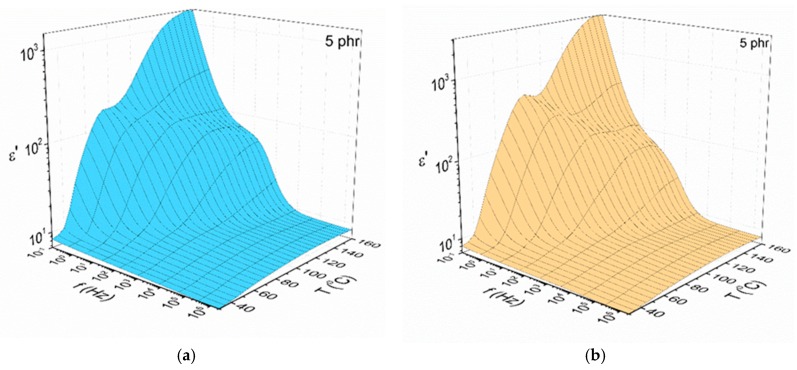
The variation of the real part of dielectric permittivity as a function of frequency and temperature for the nanocomposites with 5 phr: (**a**) BaFe_12_O_19_; (**b**) SrFe_12_O_19_ filler content.

The variation of the real part of dielectric permittivity as a function of frequency and temperature for the nanocomposites with 5 phr BaFe_12_O_19_ and SrFe_12_O_19_ concentration is presented in Figure 3a,b respectively. The dielectric permittivity diminishes rapidly with frequency, because the permanent and induced dipoles do not have the necessary time to align themselves with the externally applied electric field. The *ε*’ attains high values with increasing temperature since the thermal agitation facilitates the chain mobility thus enhancing the alignment of the chain dipoles, as well as the induced interfacial dipoles, with the field. The formation of a step like transition at intermediate frequencies and temperatures, known as α-relaxation process, is a relaxation mechanism ascribed to the glass to rubber transition of the amorphous polymer matrix. This behaviour is representative for all examined systems independent of filler type and content. 

**Figure 4:**
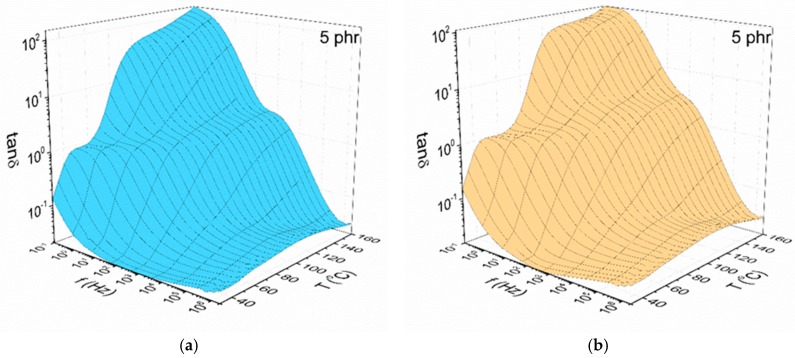
The variation of the loss tangent as a function of frequency and temperature for the nanocomposites with 5 phr: (**a**) BaFe_12_O_19_; (**b**) SrFe_12_O_19_ filler content.

Three distinct relaxation mechanisms were identified in the spectra of all examined systems as shown in Figure 4, which depicts the variation of the loss tangent versus frequency and temperature. The presence of relaxation processes becomes evident by the formation of loss peaks in the spectra of loss tangent. The faster and also weaker relaxation mechanism, appearing at high frequencies, is attributed to the reorientation of small polar side groups of the polymer chain (β-relaxation). At intermediate frequencies and temperatures, α-relaxation is recorded which is related to the transition of the polymer matrix from the glassy to rubbery state. This mechanism reflects the synergetic segmental motion of the polymer chain. Finally, at the low frequencies and high temperatures the formation of a peak is an indication of interfacial polarization (IP) or Maxwell-Wagner-Sillars (MWS) effect, which is related to the heterogeneity of the examined systems and results from the accumulation of unbounded charges at the interface between the matrix and the nanoinclusions.

## 4. Discussion

**Figure 5:**
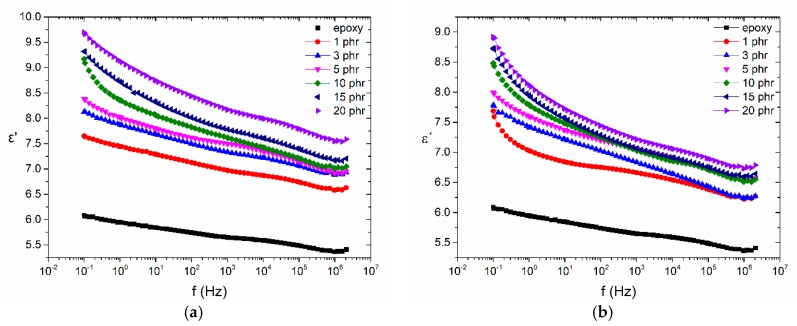
Real part of dielectric permittivity as a function of frequency, at 30 °C, for the nanocomposites with: (**a**) BaFe_12_O_19_; (**b**) SrFe_12_O_19_ nanoparticles.

Figure 5 displays comparative plots of the real part of the dielectric permittivity (*ε*’) with frequency, at 30 °C, for the (a) BaFe_12_O_19_ and (b) SrFe_12_O_19_ systems respectively. As expected, *ε*’ increases systematically with filler content in the whole frequency range, since both barium ferrite and strontium ferrite nanoparticles exhibit higher values of permittivity and conductivity than the polymer matrix. In the low frequency region, the influence of interfacial polarization due to the increasing heterogeneity of the system with filler content makes this increase more pronounced. Systems filled with barium ferrite seem to exhibit slightly higher values than those with strontium ferrite at constant filler content, in this low temperature region.

**Figure 6:**
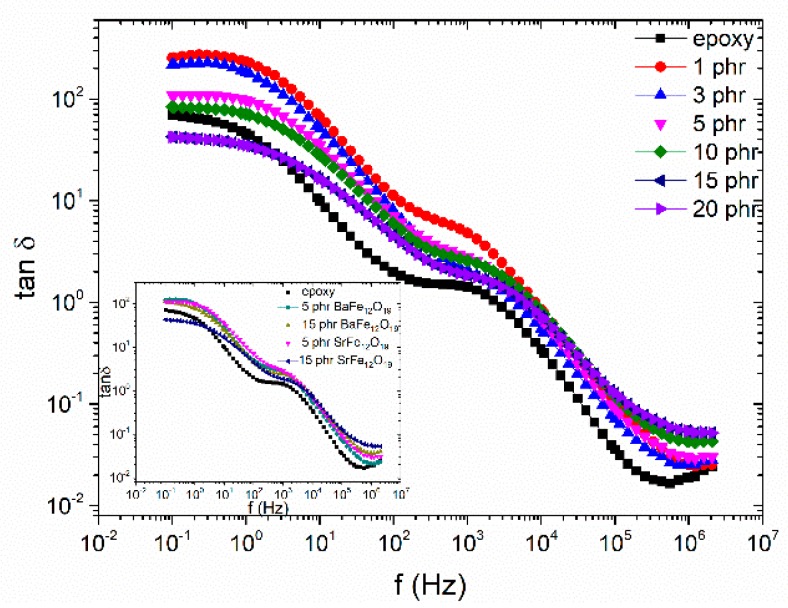
Loss tangent as a function of frequency, for SrFe_12_O_19_ examined systems, at 160 °C. Inset depicts the comparative loss tangent spectra, as a function of frequency, at 160 °C for the 5 phr and 15 phr filler content of SrFe_12_O_19_ and BaFe_12_O_19_ systems.

Comparative plots of the loss tangent, as a function of frequency, for the SrFe_12_O_19_ system, at 160 °C is presented in Figure 6. It should be noted that the barium ferrite filled systems exhibit a similar behaviour. The previously mentioned relaxations are present in these loss spectra of all studied systems. Since loss tangent is defined as the ratio of the imaginary to the real part of dielectric permittivity variations of loss tangent with filler content, in the low frequency range, should be related to interfacial polarization phenomena. Values of loss tangent diminish with barium ferrite content, reflecting the increase of polarization and thus of *ε*’, due to the enhanced heterogeneity. Interestingly, IP is observed also in the neat epoxy spectrum. IP has been detected in the loss spectra of many polymers due to the presence of additives, impurities and plasticizers. The inset diagram of Figure 6 depicts the comparative loss tangent spectra as a function of frequency at 160 °C for the 5 phr and 15 phr filler content of both examined systems. Barium ferrite systems exhibit higher values than the SrFe_12_O_19_ nanocomposites indicating that the increasing rate of the real part of permittivity is higher than the corresponding rate of the losses for the strontium ferrite filled systems. Also, the IP peaks of the strontium ferrite nanocomposites shift to higher frequencies than the barium ferrite ones indicating the facilitation of the process.

**Figure 7:**
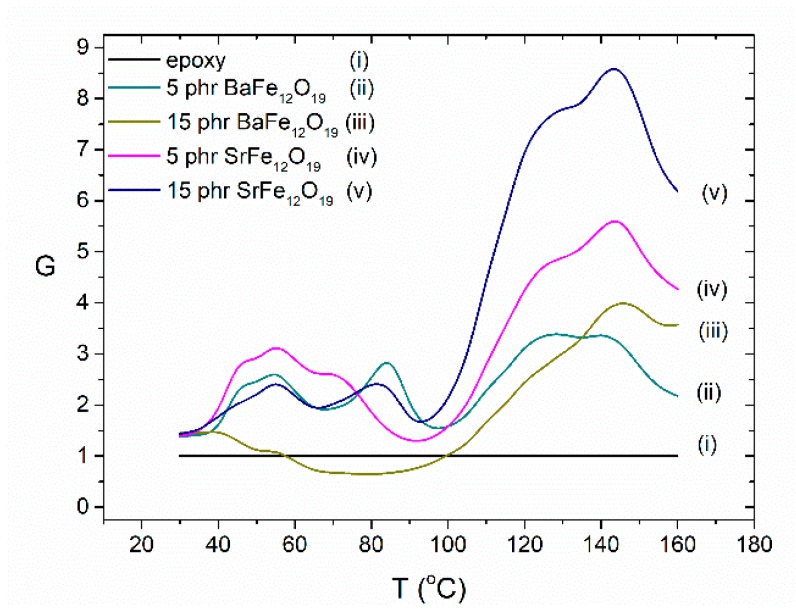
Dielectric Reinforcing Function (DRF) as a function of temperature at 0.1 Hz, for the nanocomposites with 5 phr and 15 phr BaFe_12_O_19_ and SrFe_12_O_19_ content.

Figure 7 presents a comparison of the Dielectric Reinforcing Function (DRF) as a function of temperature at 0.1 Hz for the two different fillers (BaFe_12_O_19_ and SrFe_12_O_19_) at the same concentration (5 phr and 15 phr). DRF is defined according to Equation (1):

(1) $$G\left(f,T\right)=\frac{{\varepsilon^{\prime }}_{comp}\left(f,T\right)}{{\varepsilon^{\prime }}_{matrix}\left(f,T\right)}$$

where ${\varepsilon^{\prime }}_{comp}\left(f,T\right)$ and ${\varepsilon^{\prime }}_{matrix}\left(f,T\right)$ is the real part of dielectric permittivity of the composite and the matrix respectively, while *f* is the frequency of the field and *T* the temperature. DRF is a dimensionless function being a measure of the normalized polarization, upon the geometrical characteristics of the samples, reflecting also the dielectric strengthening ability of the inclusions. So, DRF offers a strong indication relative to the energy storing efficiency of the composites [28]. The strontium ferrite systems seem to have the capability of storing more energy than the barium ferrite filled nanocomposites, even at lower filler content, in the greater part of the temperature range; reaching up to nine times the energy stored in the neat epoxy for the 15 phr specimen, at the maximum point of the function, comparing to just four times for the barium ferrite system with the same concentration. The peak formed at high temperatures is assigned to interfacial polarization because of the accumulation of unbounded charges at the interface between the filler and the polymer matrix. The shape of the peak suggests the overlapping of two or more interfacial polarization processes with different relaxation times due to the distribution of the geometrical size of the formed dipoles [16]. Microscopic results via SEM from a previous study [16] has shown the existence of two groups with different nanoinclusion sizes in the examined strontium ferrite nanocomposites. The peaks appearing as fluctuations at lower temperatures express the different dynamics of the α- and β-relaxation between the polymer matrix and the nanocomposites.

**Figure 8:**
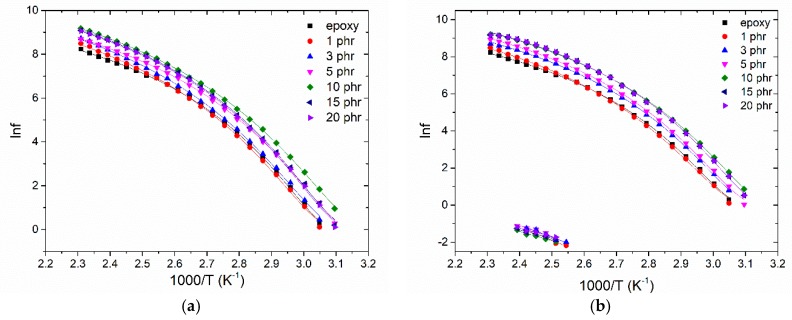
Temperature dependence of the loss peak position for the IP and α-relaxation mechanisms for the nanocomposites with: (**a**) BaFe_12_O_19_; (**b**) SrFe_12_O_19_ nanoparticles.

The relaxation dynamics or else the dependence of the loss peak frequency upon temperature for the IP and α-relaxation mechanisms for the nanocomposites with (a) BaFe_12_O_19_ and (b) SrFe_12_O_19_ nanoparticles is presented in Figure 8. The IP process for the barium ferrite filled systems as well as the β-relaxation for both examined systems, gave a limited number of loss peaks. For this reason, a reliable fitting procedure could not be performed and therefore they are missing from the study of relaxation dynamics. IP follows an Arrhenius type dependence on temperature given by Equation (2):

(2) $$f_{max}=f_{0}e^{-\;\frac{E_{A}}{k_{B}T}}$$

where *f*_max_ is the loss peak frequency, *f*_0_ a pre-exponential factor, *E_A_* the activation energy of the recorded process, *Τ* the temperature and *k_B_* the Boltzmann’s constant. The temperature dependence of the loss peak position for the glass to rubber transition process (α-relaxation) is described by the VFTH (Vogel-Fulcher-Tammann-Hesse) relation, expressed via Equation (3):

(3) $$f_{max}=f_{0}e^{-\;\frac{A}{\left(T-T_{0}\right)}}$$

where *f*_0_ is a pre-exponential factor, *A* is a measure of the activation energy of the mechanism and *T*_0_ the Vogel temperature or ideal glass transition temperature, being lower than the experimentally determined value of *T_g_*. All fitting parameters are listed in Table 1. Dielectric data can be also be employed for the estimation of the glass transition temperature via the convention that *τ (T_g,diel_*) = 100 s, which relates the relaxation time (*τ*) of α-relaxation with *T_g_* [29]. Extrapolating the VFTH curves of Figure 8 to the peak frequency, which corresponds to *τ* = 100 s, allows to determine of the *T_g,diel_* values listed in Table 1.

From the fitted parameters, apparently, all nanocomposites exhibit lower *T*_0_ and *T_g,diel_* values than the neat epoxy indicating indirectly strong interactions between the nanoparticles. The addition of nanoinclusions modifies the extend of crosslinking in the polymer system’s network, thus increasing the free volume and enhancing the chain flexibility which ultimately results in an increase of the systems’ polarization [30]. At the two higher filler contents, relaxation dynamics show an increase in the *T*_0_ and *T_g,diel_* values indicating strong adhesion of the filler to the matrix. The excess number of nanoparticles exerts spatial restrictions to macromolecules and allows the occurrence of strong attractive interactions, not only between the nanoparticles but also between nanoparticles and the polymer matrix, obstructing thus the cooperative segmental mobility of the polymer chains [16,31]. 

The activation energy of the IP process for the SrFe_12_O_19_ filled systems is listed in Table 1. Once again it should be noted that the analysis for the BaFe_12_O_19_ systems is omitted because of the limited number of peaks available for a reliable fitting. The activation energy values diminish with filler content since the increasing heterogeneity of the systems facilitates the occurrence of the IP process. The monotonous decrease of the *E_A_* values is also a sign for the fine dispersion of the nanofiller into the matrix. The formation of isolated particles’ clusters, due to agglomeration, could act as charge traps or conductive paths dead ends resulting in enhanced inertia of the dipoles at the interfaces and thus in higher relaxation times. Consequently, activation of the IP mechanism should require increased thermal agitation leading to higher values of activation energy. 

**Figure 9:**
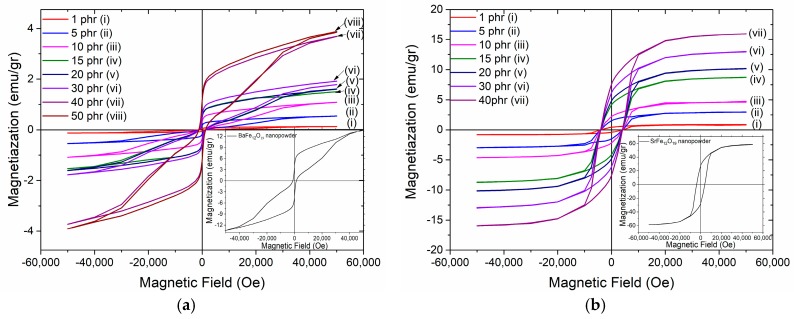
Magnetic hysteresis loops for the nanocomposites with varying: (**a**) BaFe_12_O_19_; (**b**) SrFe_12_O_19_ filler content. Insets depict the hysteresis loops of their respective nanopowders.

Figure 9a,b present the magnetic hysteresis loops, as recorded at room temperature, for polymer nanocomposites filled with BaFe_12_O_19_ and SrFe_12_O_19_ nanoparticles respectively. Each inset depicts the hysteresis loops of the respective nanopowder. The hysteresis loops confirm the ferromagnetic behaviour of nanocomposites, which is attributed to the magnetic nanoparticle content. Magnetization of the nanocomposites increases with the magnetic phase content since both barium ferrite and strontium ferrite nanoparticles induce magnetic properties into the polymer matrix. Obviously, the magnetization values of both nanocomposite systems are lower with respect to the corresponding ones of the ceramic nanopowders, due to their low filler content and the presence of the nonmagnetic polymer matrix. Coercivity values remain nearly constant for all the samples of each examined series, indicating that the coercive behaviour is controlled by the type of nanoparticles in accordance to the typical particle-loading-independent coercivity response. The coercive field, as an intrinsic property of the nanoparticles, detains the same value of 1.3 kOe for BaFe_12_O_19_ and 4 kOe for SrFe_12_O_19_ nanopowders, as well as for their respective nanocomposites. The overall shape of the hysteresis loop and the lower coercive field recorded for the BaFe_12_O_19_ nanopowder are attributed to the coexistence with the Fe_2_O_3_ phase detected in the X-ray diffraction spectra.

**Figure 10:**
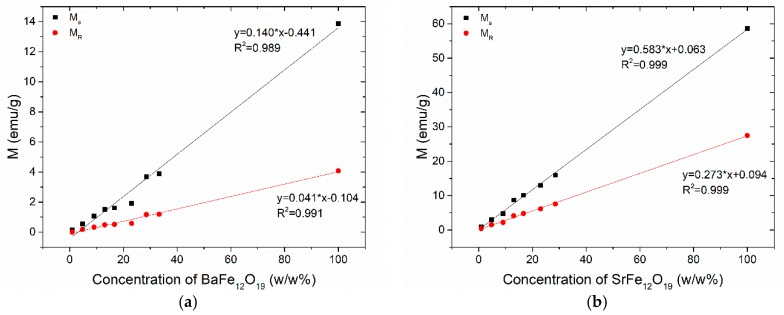
The variation of magnetization as a function of the magnetic filler concentration for the nanocomposites with: (**a**) BaFe_12_O_19_; (**b**) SrFe_12_O_19_ nanoparticles.

The variation of magnetization as a function of the magnetic filler concentration for the nanocomposites with (a) BaFe_12_O_19_ and (b) SrFe_12_O_19_ nanoparticles, determined from the hysteresis loops of Figure 9 is shown in Figure 10a,b respectively. Both the magnetic saturation (*M_s_*) and magnetic remanence (*M_r_*) of the nanocomposites increase with magnetic filler content, since the magnetic properties are induced in the nanocomposites by the included nanoparticles. The *M_s_* values of SrFe_12_O_19_ nanocomposites are approximately four times higher than those of the BaFe_12_O_19_ systems, while the *M_r_* values of the SrFe_12_O_19_ nanocomposites are even higher, approximating seven times as indicated by the slope of the linear fitting. The latter expresses the rate of increasing magnetization with filler content for each system. The observed linear increase in the values of saturation magnetization and magnetic remanence can be attributed to the fine dispersion of the magnetic nanoparticles in the polymer matrix.

**Figure 11:**
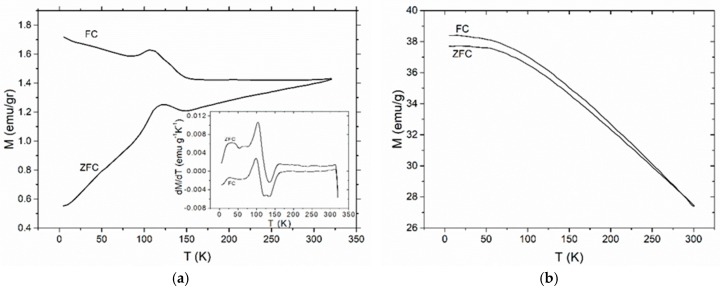
The zero-field cooled (ZFC) and field cooled (FC) magnetization curves for: (**a**) BaFe_12_O_19_; (**b**) SrFe_12_O_19_ nanopowders. Inset in (**a**) depicts the 1st derivative of ZFC and FC magnetization curves versus temperature for the BaFe_12_O_19_ nanopowder.

The low temperature magnetic properties are further elucidated through ZFC and FC magnetic measurement of the samples. Figure 11 presents the ZFC and FC magnetization measurements for the (a) BaFe_12_O_19_ and (b) SrFe_12_O_19_ nanoparticles, measured under a magnetic field of 1000 Oe. Figure 11 shows the ZFC and FC magnetization measurements for the nanocomposites with 5 and 40 phr content in (a) BaFe_12_O_19_ and (b) SrFe_12_O_19_, measured under a field of 100 Oe. The FC magnetization curve is always above the ZFC curve up to room temperature. The irreversibility temperature (*T*_irr_) or else the temperature where the ZFC splits from the FC curve, is at 300 K for both the nanocomposite systems and their respective nanopowder, which is characteristic for non-interacting magnetic particles.

**Figure 12:**
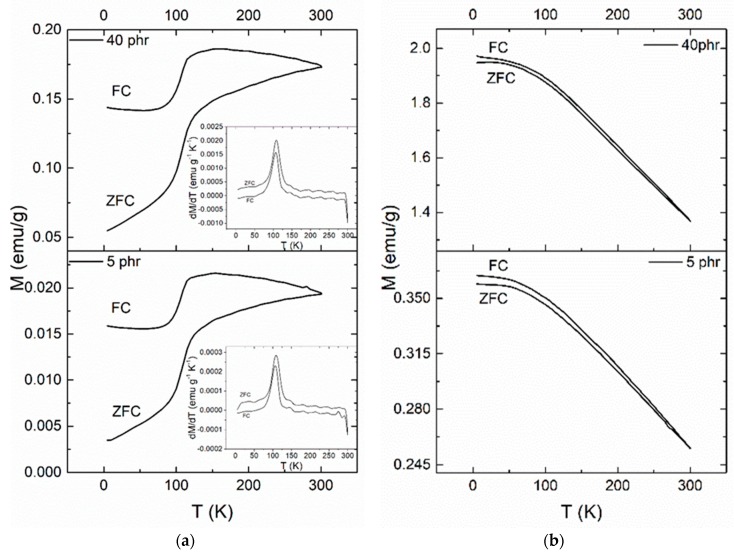
The ZFC and FC magnetization curves for the 5 and 40 phr nanocomposites with: (**a**) BaFe_12_O_19_; (**b**) SrFe_12_O_19_ filler content. Inset in (**a**) depicts the 1st derivative of the ZFC and FC magnetization curves versus temperature for the 5 and 40 phr BaFe_12_O_19_ nanocomposites.

The ZFC curve of barium ferrite and its nanocomposites increases with temperature forming a peak approximately at 110 K, which is followed by a slow increase of magnetization up to 300 K. This behaviour signifies a magnetic transition. In order to further investigate this behaviour the first derivative of ZFC and FC curves has been calculated for the barium ferrite systems. Representative plots are depicted as insets in Figure 11a and Figure 12a. Peak’s position was found to be at 109 K in all nanocomposites with barium ferrite and at 105 K for the neat nanopowder. This small difference could be assigned to the stronger employed field for the nanopowder measurement. Moreover, peaks in FC curves seem to slightly shift to lower temperatures. Recorded transition cannot be easily interpreted and attributed undoubtedly to a specific physical mechanism. The simultaneous presence of barium ferrite and iron oxide in the reinforcing nanopowder necessitates examining both ingredients as the possible origin of the transition. The peak of the detected transition appears at a temperature closed to the Verwey transition (occurring at *T_V_* ~ 120 K) of Fe_3_O_4_, which is a first order magnetic phase transition related to the change of magnetocrystalline anisotropy and ordering of Fe^3+^ and Fe^2+^ ions at the octahedral sites of cubic spinel structure. Verwey transition is considered as characteristic of a pure, nearly stoichiometric population of magnetite. Under oxidative conditions magnetite transforms gradually to its oxidized form of maghemite (*γ*-Fe_2_O_3_). In the literature there are studies reporting that the presence of non-stoichiometric maghemite induces a decrease in Verwey temperature [32,33,34]. Other studies about polycrystalline Ba hexaferrites and BaO*nFe_2_O_3_ of slightly deviating stoichiometry, have also indicated the occurrence of this process with decreasing *T_v_* temperature [35,36,37]. On the other hand, the polymorphism of iron oxides and the low magnetization values of BaO*6Fe_2_O_3_ in tandem with its difficulty to reach saturation (inset of Figure 9a) suggests another interpretation by considering that the present iron oxide could be, at least partially, in the form of *α*-Fe_2_O_3_ (hematite) or *ε*-Fe_2_O_3_. In the case of *α*-Fe_2_O_3_, in its bulk form, a magnetic transition from the weak ferromagnetic phase to antiferromagnetic one occurs at 260 K, known as Morin temperature (*T_M_*). Morin temperature appears to be sensitive on particle shape, size, crystallinity and in general decreases with particle size, vanishing for spheroids with diameter below 10 nm [38,39,40]. Furthermore, the *ε*-Fe_2_O_3_ polymorph of ferric oxide exhibits a magnetic transition at approximately 110 K. *ε*-Fe_2_O_3_ exists only in nanoscale and transforms to hematite upon increasing its dimensions and by heating [41,42,43,44]. Unconfined *ε*-Fe_2_O_3_ nanoparticles transform to hematite at temperatures ranging between 700 and 1300 K [41]. At 110 K *ε*-Fe_2_O_3_ undergoes a transition from a magnetic ordered state to another magnetic status. A dispute for the physical origin of this transition is present in the literature. The first approach considers a magnetic transition from a collinear ferromagnetic state to an incommensurate magnetic structure (probably of a square-wave-modulated origin). The second one, considers the transition from a canted antiferromagnetic state (characterized by a specific canting angle) to another canted antiferromagnetic state (characterized by a different canting angle). The latter resembles to a Morin-like transition [41,42,43].

In order to elucidate the possible origin of the recorded transition in Figure 11a and Figure 12a a detailed analysis of the relative XRD spectra was carried out. For this reason, suitable software was employed (Match! Crystal Impact, Bonn, Germany). Results indicate the presence of at least two phases. The first one has an hexagonal crystal system with the *P6_3_/mmc* space group and lattice parameters *a* = 5.8000 Å and *c* = 23.1800 Å and is attributed to BaFe_12_O_19_, while the second one has an orthorhombic crystal system with the *Pna2_1_* space group and lattice parameters *a* = 5.0850 Å, *b* = 8.7740 Å and *c* = 9.4680 Å and is attributed to *ε*-Fe_2_O_3_. Analysis shown also the existence of a limited number of peaks which cannot be accurately identified. These peaks could be related to the *α*-Fe_2_O_3_ and *γ*-Fe_2_O_3_ polymorphs of iron oxide, which might be present in limited quantities, in the nanopowder. At this point it should be noted that the preparation methods of barium ferrite lead to the formation of residual phases which include iron oxide polymorphs [45,46]. Summarizing the above discussion, it can be concluded that the recorded magnetic transition cannot be attributed without any doubts to a specific physical mechanism and more research should be conducted in this direction. However, combining XRD spectra with the results from the ZFC and FC curves the recorded magnetic transition it is possible to originate from the *ε*-Fe_2_O_3_ polymorph. Finally, to the best of our knowledge, this is the first time where a magnetic transition is observed in ferrite/polymer nanocomposites.

## 5. Conclusions

Two different series of nanocomposites consisting of epoxy resin and BaFe_12_O_19_ or SrFe_12_O_19_ nanoparticles were successfully fabricated and characterized morphologically via scanning electron microscopy and X-ray diffraction spectra. The dielectric response of the nanocomposites was investigated by means of broadband dielectric spectroscopy and their magnetic properties were derived from data obtained by a superconducting quantum interference device. Analysis of the experimental data implies that fine dispersion of the ceramic nanoparticles in the polymer matrix enhances substantially both the magnetic and the dielectric performance of the investigated systems. Although the examined systems exhibit similar behaviour, SrFe_12_O_19_ nanocomposites seems to have the optimum dielectric behaviour in the greater part of the experimental range attaining higher values of *ε*’ than the BaFe_12_O_19_ nanocomposites. Dielectric spectra of all examined systems revealed the presence of three distinct relaxation mechanisms which are attributed to: (i) Interfacial polarization, also known as Maxwell-Wagner-Sillars effect, observed in the low frequency and high temperature region because of the heterogeneity of the systems, (ii) α-relaxation, present at intermediate temperatures and frequencies, related to the glass to rubber transition of the polymer matrix and (iii) *β*-relaxation due to the re-arrangement of small polar side groups of the macromolecular chain at high frequencies. The magnetic measurements confirmed the ferromagnetic nature of the nanocomposites. The induced magnetic properties increase with the inclusion of hexaferrite nanoparticles. The nanocomposites with SrFe_12_O_19_ nanoparticles exhibit higher values of coercive field, magnetization, magnetic saturation and remanence magnetization. The ZFC/FC tests for the barium ferrite systems revealed an interesting behaviour due to the detected magnetic transition at a characteristic temperature of approximately 109 K.

## Figures and Tables

**Table 1:** Specimens’ composition in phr, activation energy values and parameters derived via fitting data with Equations (2) and (3), for all studied systems and glass transition temperature as determined via BDS.

Specimens	BaFe_12_O_19_			SrFe_12_O_19_			
	α-Relaxation		*T_g, dielectric_* (K)	α-Relaxation		IP	*T_g, dielectric_* (K)
	*A* (K)	*T*_0_ (K)		*A* (K)	*T*_0_ (K)	*E_A_* (eV)	
Epoxy	17.5	315.9	320.9	17.5	315.9		320.9
1 phr	19.9	314.9	320.7	19.3	314.7	0.624	320.2
3 phr	20.5	312.3	318.1	18.6	312.1	0.571	317.4
5 phr	18.1	310.3	315.4	18.3	311.7	0.526	316.9
10 phr	20.1	304.7	310.3	18.3	307.1	0.427	312.3
15 phr	18.3	310.5	315.7	17.4	309.6	0.399	314.5
20 phr	18.4	311.0	316.2	17.4	309.6	0.376	314.5
